# A survey of women who continue to smoke during pregnancy in Slovenia

**DOI:** 10.18332/ejm/95283

**Published:** 2018-10-02

**Authors:** Tjaša Rozman, Polona A. Mivšek, Anita Jug Došler, Mateja Kusterle

**Affiliations:** 1 Faculty of Health Sciences, University of Ljubljana, Ljubljana, Slovenia; 2 Slovenian Institute for Adult Education, Ljubljana, Slovenia

**Keywords:** health education, pregnancy, smoking cessation, pregnant women, knowledge

## Abstract

**INTRODUCTION:**

Smoking in pregnancy is unhealthy and can also have negative effects on the foetus. However, there are still some women who do not quit smoking during pregnancy. The aim of the study was to identify Slovenian women at risk who smoke during pregnancy.

**METHODS:**

A quantative approach using an online survey was adopted. The study was conducted in May and June 2017, in Slovenia. A snowball sample was used. Participation in the survey was anonymous and voluntary, and 118 women who identified themselves as smokers participated in the study. Descriptive statistics was used to analyse the data.

**RESULTS:**

More than half (66%) did not quit smoking during pregnancy. Women who continued to smoke during pregnancy were usually younger, less educated with a partner who smoked. Women who smoked more cigarettes per day before conception were less likely to refrain from smoking during pregnancy. The most common reason for not quitting smoking during pregnancy was failure in attempts to quit (37%).

**CONCLUSIONS:**

Women who smoke need support to stop smoking before pregnancy or, at least, in the first trimester. Special groups for smoking cessation need to be created. Further and more extensive research is warranted in Slovenia to assess this issue.

## INTRODUCTION

The proportion of smokers is declining in Slovenia, nevertheless approximately 24% of the population smoke^1,2^. Rates are higher for men than for women. In Slovenia, smoking is prohibited by law in public places and the workplace since 2001, and therefore employees have no work breaks for smoking^3^. No number of cigarettes during pregnancy is considered safe. Each cigarette that a pregnant woman smokes exposes her child to harmful effects for 15 minutes^4^. Smoking is a bad habit that pregnant women should stop, unfortunately research shows that some do continue. In 2010, a research study conducted on a sample of pregnant Slovene women recorded 10.4% of selfproclaimed smokers, of these 71.2% did not give up smoking while 28.8% did^5^. More recent data from Spain^6^ reveal that of 29.7% pregnant smokers, 41.4% spontaneously quit in the first trimester while 58.6% continued to smoke. US statistics^7^ show that up to 40% of women quit smoking during pregnancy; however, more than half relapsed within 6 months and up to 90% relapsed within a year.

Slovenian researchers^5^ found that pregnant women who smoke gain about 4 kg more, on average, during pregnancy, associated with their inactive lifestyle. O’Malley et al.^8^ claim that smoking has an impact on taste perception that results in different dietary intakes of macro/micro nutrients among smokers and non-smokers. Their research revealed that pregnant women who smoke have a poor nutritional intake relative to non-smokers. In particular, they have lower levels of magnesium, iron, carotene, copper, vitamin C, riboflavin, folate and fibre, but higher levels of sodium and chloride that may lead to dietary deficiencies. Bush et al.^9^ showed that about 75% of smokers gain weight after quitting, without other permanent lifestyle measures.

Women who smoke during pregnancy are usually younger^10-12^, less educated^5,12^, unemployed^11,12^, single^1,2^ and heavy smokers^13^. Moreover, they often feel helpless, sensitive and vulnerable^5^, possibly due to a predisposition to mental health issues, as described in current literature^14,15^ on the relationship between smoking and depression or anxiety.

A meta-analysis of eighty-five studies^14^ revealed that smoking is associated with a nearly two-fold increased risk of depression relative to never smokers and former smokers, with the association supposedly stronger in women than in men. A longitudinal study^15^ performed in the US, from 2005 to 2014 observed changes in the prevalence of cigarette smoking among pregnant women with and without a major depressive episode from the year preceding. A conclusion was that smoking during pregnancy increased in the group of women who experienced a major depressive episode at that time (from 35.9% to 38.4%) and decreased in the remaining group of smoking pregnant women (from 12.5% to 9.1%). Overall, smoking appeared to be over four times more common among pregnant women with a major depressive episode compared to those without. The vulnerable group consisted of younger women, widowed, separated, divorced or never married, with a low income and other children. Such results emphasize the need for a deeper understanding of the relationship between mental health and prenatal smoking, in order to develop effective targeted smoking cessation interventions.

Active smokers experience complications in pregnancy more frequently (73.3%) than non-smokers (16.7%)^14^. Smoking increases the likelihood of complications in pregnancy, such as miscarriage^16,17^, placenta praevia^18^ and placental abruption^19^, preterm labour, premature rupture of membranes^16,20^ and the need for a caesarean section; it also increases the risk of breech presentation, lower Apgar score of the newborn in the first and fifth minute after birth, and the risk of foetal distress^16^. The risk of preterm labour is increased in women who smoke in the second and third trimesters of pregnancy, while women who quit smoking in the first trimester have a similar risk as non-smoking pregnant women^21^. Research shows that newborns of mothers who smoke in the first trimester of pregnancy are 20–70% more likely to have congenital heart defects, whereas cessation of smoking before the onset of pregnancy or very early in pregnancy might prevent these^16,22^. Researchers^23^ have discovered that smoking during pregnancy increases the risk of a split lip or cleft palate, low birth weight in the newborn^3,20^ and risk of being stillborn for 47%^24^. A complication frequently associated with babies of smoking mothers in the first year of life is Sudden Infant Death Syndrome SIDS^25,26^, where the cause of death is uncertain^27^.

Due to the relatively high percentage of women who smoke during pregnancy in Slovenia^5^, the present study researched the most common reasons for quitting or smoking in pregnancy. The purpose was to study the reasons behind smoking behaviours of women in pregnancy and to define the characteristics of women who smoke during pregnancy, in order to organise future midwifery care tailored to the vulnerable groups of women who need additional help to quit the habit. We posed three research questions: 1) Which women are more likely to continue smoking during pregnancy?, 2) What is the most frequent reason for smoking cessation during pregnancy?, 3) What is the most frequent reason to continue smoking during pregnancy?

## METHODS

A non-experimental method of empirical research was applied, supported by a survey as the data collection technique. The research instrument was a survey questionnaire that included 16 questions of either closed-ended type or combined closed and open-ended type. The lead author of the article designed the questionnaire and included an online tool. The pilot phase intended to test the clarity of the questions was launched on 10 May 2017 and involved ten participants. The pilot testing took place on a social network until 11 May 2017. Since no corrections to the questionnaire were required, the ten preliminary questionnaires were included in the primary data analysis.

The survey questionnaire that included an informed consent to participate in the study was published on a social network and forwarded by e-mail. Snowball sampling was used. The survey was active for 11 days from 28 May to 7 June 2017. Participation in the survey was anonymous and voluntary. The sample included 118 women who were or still are active smokers and were pregnant at least once.

The online survey ensured the anonymity of participants. The study was approved by the Cathedra of Midwifery, Faculty of Health Sciences Ljubljana, Slovenia. Participation was voluntary.

The collected data were analysed and statistically evaluated with the help of a tool for online surveys. The analysis calculated basic descriptive statistics with frequencies and percentages. The results were compared with other current international research.

## RESULTS

Based on the inclusion criteria of the study, 118 women participated who were active smokers or smoked, and who were pregnant at least once. Of these, 66 respondents, i.e. more than a half, smoked during pregnancy, while 52 had quit.

Five pregnant women were 20 years old or younger. In this group, 4 continued to smoke. Of 90 women pregnant at 21 to 30 years of age, 39 women (43.3%) quit smoking in pregnancy, and 51 women (56.6%) did not. Of 23 women pregnant at 31 to 40 years of age, smoking was abandoned by 12 (52.2%), whereas 11 (47.8%) continued to smoke. Women older than 40 years did not participate in the study.

The question of educational attainment at the time of pregnancy was answered by 117 women (Table 1). None of the women who completed primary and lower-secondary education stopped smoking; 2 women continued to smoke. Of the 14 women who completed vocational upper-secondary education, 11 continued to smoke in pregnancy, whereas 3 women quit. Of 53 women who completed general uppersecondary education, 21 quit smoking and 32 continued the habit.

**Table 1:** Educational attainment during pregnancy of non-smokers and smokers in Slovenia, 2017 — percentage values (N=117)

*ANSWERS*	*Non-smokers (44%)*	*Smokers (56%)*
(Un)completed primary and lower-secondary education (1.7)	0.0	100.0
Vocational upper-secondary education (12)	21.4	78.5
General upper-secondary education (45.3)	39.6	60.4
Higher vocational, professional and academic education (37.6)	59.1	40.9
Master's, specialization, doctorate (3.4)	50.0	50.0

Among 44 women who completed higher vocational, professional and academic education, 26 quit smoking during pregnancy and 18 continued to smoke. Of the 4 women with a Masters degree, a specialisation, or a Doctoral degree, two quit smoking.

In all, 117 women answered the question on marital status. Only one woman was single during pregnancy and did not quit smoking. Of 32 married women, 20 (62.5%) quit and 12 (37.5%) continued to smoke. Among 84 women who were in a cohabiting partnership whilst pregnant, 32 (38.1%) quit smoking, whereas 52 women (61.9%) continued to smoke. No divorced woman participated in the study.

The question whether the woman’s partner smoked during her pregnancy was answered by 116 women. In 71 relationships where both partners smoked, only 25 women (35.2%) quit smoking, whereas 46 women (64.8%) continued. In the group of 45 pregnant women whose partners did not smoke at the time, 27 women (60%) quit and 18 (40%) continued to smoke.

The question of employment status in pregnancy was answered by 117 women. Among 78 pregnant women who were employed, 34 (43.6%) quit smoking and 44 (56.4%) continued. Among five women who were self-employed at the time of the pregnancy, 3 (60%) continued to smoke. The research study included 19 women who were unemployed during pregnancy, and among them, 10 (52.6%) quit smoking and 9 (47.7%) continued. Fifteen women who participated in the study were students in secondary or tertiary education while pregnant. Six (40%) quit smoking and 9 (60%) did not. Women who worked as housewives during pregnancy did not participate in the study.

### Health awareness and smoking cessation

The question on the number of cigarettes smoked daily before conception was answered by 116 women. All women who smoked fewer than one cigarette daily (N=14) before conception quit the habit during pregnancy. Among the women who smoked 1 to 5 cigarettes daily (N=15) before conception, ten pregnant women (66.6%) quit and 5 (33.3%) continued. Among the women who smoked 6 to 10 cigarettes daily (N=25) before conception, 12 pregnant women (48%) quit smoking, and 13 pregnant women (52%) did not. In the group of women who smoked between 11 and 15 cigarettes daily (N=28) before conception, only nine of the pregnant women (32.1%) quit the habit whereas 19 women (67.9%) continued. Among the women who smoked 16 to 20 cigarettes daily (N=24) before conception, as few as five women (20.8%) stopped smoking in pregnancy and 19 (79.2%) continued. In the group of women who at pre-conception smoked more than 20 cigarettes daily (N=10), only one woman (10%) quit and nine women (90%) continued while pregnant.

Among the women whose pregnancy was wanted and planned (N=72), 37 women (51.4%) gave up smoking in pregnancy whereas 35 women (48.6%) continued. In the group of women whose pregnancy was wanted but unplanned (N=42), 14 women (33.3%) stopped and 28 women (66.6%) continued during pregnancy. The study included only one woman who had an unwanted, but planned pregnancy, but did not quit smoking. Likewise, one woman who participated in the study had an unwanted, unplanned pregnancy and did not quit smoking.

Among those who supplemented folic acid in pregnancy (N=100), 46 women (46%) quit smoking and 54 women (54%) continued. The women who did not add folic acid to their diet during pregnancy (N=16) stopped smoking in 5 cases (31.3%) while 11 (68.7%) continued the habit.

In the group of women who had the first pregnancy appointment with gynaecologist before 12 weeks of pregnancy (N=100), 47 women (47%) quit smoking whereas 53 women (53%) did not. Among the women who had the first pregnancy appointment with gynaecologist after 12 to 20 weeks of pregnancy (N=15), only 4 (26.7%) quit smoking and 11 (73.3%) continued.

### Reasons for smoking cessation in pregnancy

The question concerning the reasons for smoking cessation during pregnancy was answered by 50 women smokers who quit the habit before or during that time. They could choose more than one answer. All opinions were gathered and ranked (Table 2).

**Table 2:** The most frequent reasons for smoking cessation during pregnancy in Slovenia, 2017 — percentage values (N=50)

*ANSWERS*	*%*
Awareness that smoking is harmful to the child.	37
The desire to provide the child with optimal conditions for development.	33
I had no desire to smoke during pregnancy.	15
Other: I quit smoking several years before becoming pregnant.	9
I was worried what other people would think when they saw a pregnant woman smoking.	3
The desire of my partner and my close ones to quit smoking.	2

Thirty-two women quit smoking due to awareness that smoking is harmful to the child, and 29 women gave up smoking because they wanted to provide the child with optimal conditions for development. Thirteen women did not smoke during pregnancy because they felt no desire to do so.

### Reasons to continue smoking in pregnancy

The question concerning the reasons why they continued to smoke during pregnancy was answered by 64 women smokers. They could choose more than one answer. All opinions were gathered and ranked (Table 3).

**Table 3:** The most frequent reasons for smoking during pregnancy in Slovenia, 2017 — percentage values (N=64)

*ANSWERS*	*%*
I tried to quit smoking, but I failed.	36
Stress I experienced during pregnancy.	16
Knowing of other pregnant women and mothers who smoked during pregnancy and gave birth to a healthy child without any consequences of smoking.	16
I saw no reason to quit smoking in pregnancy.	8
I did not want to quit smoking.	8
Other: my gynaecologist advised against smoking cessation during pregnancy because it could harm the baby.	7
Smoking of my partner and/or close ones.	7
I lacked support to quit smoking.	4

The common reason for thirty-two women was that they tried to quit smoking but failed. None of the women answered that the smoking of their friends influenced them to continue to smoke in pregnancy. For 14 pregnant women, the principal reason was the stress they experienced at the time. Fourteen women reasoned that their choice was based on knowing other pregnant women and mothers who smoked during pregnancy and gave birth to a healthy child, without any consequences.

## DISCUSSION

The results reveal that 56% of the respondents remained active smokers during pregnancy. It corresponds with the findings of previous studies: a Slovenian study, where among 10.4% of active smokers as many as 71.2% continued to smoke in pregnancy^5^; a Spanish^6^ study, where 58.6% continued to smoke; and a US study with a 60% continuation rate^7^. The result of the present study is rather alarming because it shows that every other woman smoker continues to smoke during pregnancy, despite all the risks the habit poses to the pregnancy and the foetus.

In reference to the age of pregnant women smokers, the present study reveals that the percentage of pregnant women who continue to smoke is higher among younger women, which corresponds with the findings of other studies^6,7,10,11,12^. Regarding education, the women with lower levels of education usually remain smokers in pregnancy compared to women with higher levels of education, confirmed by earlier studies^5,6,7,12^. The findings indicate that women with a higher degree of education are more aware of the risks of smoking during pregnancy, and follow medical advice and recommendations to a greater extent.

The percentage of failure to quit smoking during pregnancy was higher in the groups of single women and women who were in a cohabiting partnership, compared to pregnant married women. Furthermore, the percentage of smoking pregnant women was higher for those in relationships with an active smoker partner, as confirmed by previous studies^6,12^. This leads us to conclude that smoking partners of pregnant women have a negative impact on her smoking cessation attempts. Therefore, it would be reasonable to develop programmes for smoking cessation that would include both partners. Joint effort to quit smoking would enable mutual support and increased motivation between partners, leading to a higher success rate.

The women’s employment status during pregnancy also influenced the smoking rate at that time. More women who were employed or self-employed during pregnancy continued to smoke compared to unemployed pregnant women and students in secondary or tertiary education. The results are discordant with other research^11,12^, which concluded that the percentage of smoking pregnant women was lower in the permanently employed and higher in the unemployed. Our findings could be explained based on the power to purchase cigarettes. Some researchers mention stress^15,28^, anxiety and depression^6,29^ that might be exacerbated by being employed. More highly educated women with a college degree or higher often fall into this category, despite their knowledge of the harmful effects of smoking^15^. Research shows that they might smoke to improve their immediate sense of well-being or as a quick reward^28^. The present study, unfortunately, did not cover these aspects of smoking. Based on the number of cigarettes smoked daily before conception, the percentage of pregnant smokers increased with the number of cigarettes, as found by other studies^6,13^. Smoking cessation also depended on whether the pregnancies were wanted and planned. Women who planned their pregnancy were better motivated for a healthy lifestyle, self-care and care for the unborn child. Our study shows that the percentage of women whose pregnancy was wanted and planned but did not quit smoking was considerably lower than for those with unplanned and unwanted pregnancies. A healthy lifestyle, self-care and care for the foetus may surface in different ways. Accordingly, our research showed that many pregnant women who smoked also did not take supplements of folic acid and had the first appointment with gynaecologist after 12 weeks of pregnancy, consistent with previous research^10,11^.

The findings above lead to the conclusion that women who want and plan their pregnancy also take into account medical advice and recommendations. We have found that a common motivation for smoking cessation during pregnancy is awareness that smoking is harmful to the baby. We, therefore, deduce that women who stop smoking in pregnancy place the well-being of their child before their own pleasure, in order to protect it from the negative influences of smoking while in the womb.

It is essential to distinguish the reasons for continuation and cessation of smoking in order to develop tailored interventions and appropriate counselling to pregnant women. The most frequent reason, given in our study, why women did not quit smoking in pregnancy was that they attempted to quit but failed. The question arises whether the failure to quit was a consequence of nicotine addiction or a lack of support to quit smoking. It would be reasonable to introduce pilot support groups for women who plan to get pregnant, and for pregnant women and their partners, to offer appropriate information on smoking in pregnancy and continuous support.

It is alarming that 8% of women did not see a reason to quit smoking during pregnancy, as found also by Polen et al.^29^ for 5% of the women. It would thus be sensible to upgrade the study with a qualitative approach and examine the reasons for not quitting smoking; it might have been ignorance of the consequences of smoking; possibly it was the women’s conscious decision; or they developed such a severe addiction that they lacked the self-confidence to succeed. The last could be true for the women with depression or anxiety symptoms^6^ because nicotine withdrawal induces an even more dysphoric mood, insomnia, irritability, anxiety and frustration^28^. De Wilde et al.^28^ discovered that pregnant smokers perceived smoking as an essential coping mechanism for tension and stress reduction. Other prevalent reasons were pleasure and addiction, whereas habit and social function were the least important. Polen et al.^29^ report that many women have a good knowledge of the adverse effects of smoking in pregnancy but do not quit due to lack of motivation.

An interesting piece of information was that 7% of the respondents of the present study also listed as a reason to continue smoking the view that stopping would harm the baby, and thus the gynaecologist advised against smoking cessation during their pregnancy. Smoking cessation recommendations encourage women to quit smoking entirely in the first trimester of pregnancy, and if they do not succeed to at least gradually reduce the number of cigarettes smoked daily and to completely abandon smoking in the third trimester^27^. It is unclear if the gynaecologists did in fact offer such advice, or whether it was interpreted incorrectly by the pregnant women.

The main limitation of the study is that the online survey did not enable any control over the participants, and therefore the sample may not be representative. On the other hand, an online survey gives participants sufficient feeling of anonymity to discuss socially unwanted behaviours, such as smoking during pregnancy. We decided to use this kind of tool primarily due to the anonymity since, in the questionnaires delivered by the health professionals, respondents often give socially expected answers. In Slovenia, there are approximately 20000 births per year^30^. The last estimation^5^ of the percentage of women who smoke during pregnancy was made in 2010. There it was found that about 10% of women continue to smoke during pregnancy (about 2000 women per year). We cannot claim that the sample of 118 women included in the present survey can, therefore, be representative; however, it does offer valuable information on the subject. The study^5^ conducted in 2010, however, did neither focus on the characteristics of women who smoke in pregnancy nor on the reasons why women do not quit smoking in pregnancy and the target national actions appropriate for them. In that sense, our study, despite its methodological weaknesses mentioned above, is original and provides valuable insights.

## CONCLUSIONS

We can conclude that women in Slovenia should receive information on the adverse effects of smoking during pregnancy in time and even before conception. Smoking cessation before or during pregnancy influences the course of the pregnancy as well as foetal development. More attention should be given to women identified, by previous studies and the present, as vulnerable to smoking (younger, less educated, single, heavy smokers, partners smoking) to assist them to have greater smoking cessation success.

Since pregnant smoking women feel anxious about joining general support groups for smoking cessation (they are afraid of stigma), special groups should be created for them. Studies have shown that because more women who are cohabiting with smoking partners continue to smoke in pregnancy, support groups should actively involve both partners in the process of smoking cessation. Women should be screened for possible psychological problems, which should be separately addressed and taken into consideration in smoking cessation programmes.

Many women in the study reported that they attempted to quit, but failed. Therefore, we conclude that pregnant women receive insufficient support during this vulnerable period. Formation of support groups and self-help groups for smoking cessation, intended exclusively for women who are planning a pregnancy or are pregnant, would enable women to confront the issue more discretely as they would only join couples who face similar problems. Furthermore, in the future, it would be reasonable to carry out qualitative research on the women who did not quit smoking in pregnancy, as this would offer a deeper understanding of the motives and reasons for their decision-making that might lead to a more in-depth insight and opportunities for planning further measures. Overall, our research demonstrates a need for greater health education about smoking in pregnancy in Slovenia, a need to develop services for smoking cessation especially tailored to vulnerable groups of women planning a pregnancy, and a need for more significant support of the couple as a unit, since the lifestyle of the partner also affects the woman’s decisions.

## CONFLICTS OF INTEREST

Authors have completed and submitted the ICMJE Form for Disclosure of Potential Conflicts of Interest and none was reported.
